# Exploring intimacy and family planning in Inflammatory Bowel Diseases: a qualitative study

**DOI:** 10.1080/07853890.2024.2401610

**Published:** 2024-11-18

**Authors:** Kristyna Gabova, Hana Bednarikova, Zdenek Meier, Peter Tavel

**Affiliations:** Olomouc University Social Health Institute, Univerzita Palackeho v Olomouci, Olomouc, Czech Republic

**Keywords:** IBD, sexuality, family planning, ulcerative colitis, Crohn’s disease

## Abstract

**Background:**

Inflammatory bowel diseases (IBD), comprising ulcerative colitis and Crohn’s disease, have a profound impact on people’s lives. This study aims to investigate the perceived impact of IBD on sexual life and family planning to enhance understanding of the interplay between IBD, sexuality, intimacy and family planning to foster a better quality of life for those living with the condition.

**Materials and methods:**

The study used the qualitative DIPEx method (Database of Personal Experiences of Health and Illness) developed by the Health Experiences Research Group at the University of Oxford, standardized for the Czech Republic. Semi-structured interviews with 36 participants (19 with Crohn’s disease, 17 with ulcerative colitis) were conducted, employing open coding and thematic analysis. The maximum variation sampling was utilized, considering various factors, such as disease stage, medications, treatments, age, age at diagnosis and sociodemographic characteristics.

**Results:**

Five main topics emerged: sexual activity, body image and discomfort, partner relationships, family planning, and the role of gastroenterologists in family planning decisions. Participants’ perceptions of sexuality varied, with some experiencing reduced sexual activity during acute phases of the disease. IBD had contrasting effects on partner relationships, and family planning was an important concern, with women valuing support and collaboration with their gastroenterologists in making pregnancy decisions. Information dissemination and open communication from professionals are highly needed.

**Conclusions:**

This is the first study concerning the sexuality and family planning of people with IBD in the Czech Republic. It highlights the need for information and open communication from professionals.

## PLAIN LANGUAGE SUMMARY

Intimacy and Family Planning in Inflammatory Bowel Diseases. This study focuses on understanding how Inflammatory Bowel Diseases (IBD) like ulcerative colitis and Crohn’s disease affect personal aspects of life such as sexual activity, relationships, and decisions about starting a family. Through interviews with 36 individuals living with IBD, researchers found that these conditions can significantly impact one’s sex life, how they see themselves, their comfort with their bodies, and their relationships with partners. It also explored how people with IBD make decisions about having children, often needing more guidance and open conversations with their doctors. The findings emphasize the importance of clear communication and support from healthcare professionals to improve the quality of life for those with IBD. This research is a first of its kind in the Czech Republic.

## Graphical abstract

**Figure:**
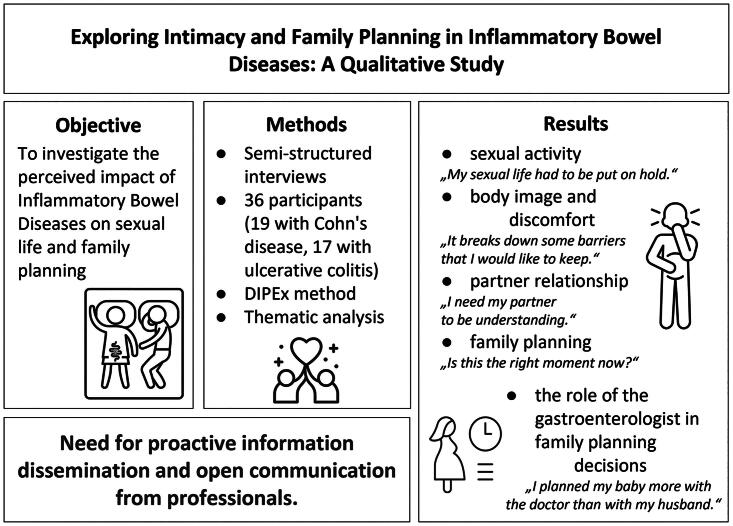

## Introduction

Inflammatory bowel diseases (IBD) represent a diverse variety of chronic inflammatory intestinal conditions, with two major types: ulcerative colitis (UC) and Crohn’s disease (CD). IBD can manifest with various symptoms and complications, such as diarrhoea, blood in the stool, pain, nausea, incontinence, weight loss, flatulence and fatigue [1,2]. There is evidence of an interaction between psychological factors and IBD [3–6]. This may lead to consequences that could affect sexual life, body image, intimacy and sexual function [7–12]. However, it is alarming that the topic of sexuality is often neglected in the healthcare dialogue [12]. The importance of the topic is further underscored by the European Crohn’s and Colitis Guidelines on Sexuality, Fertility, Pregnancy, and Lactation [11]. The complex interplay between IBD and sexuality necessitates understanding and addressing the concerns related to sexual well-being and intimate relationships in individuals with IBD. This research is the first study concerning the sexuality and family planning of people living with IBD in the Czech Republic.

### Background

Individuals with IBD often find themselves at their peak conceiving period or at a stage in life where sexual identity becomes essential. This makes addressing concerns about sexuality, intimacy and family planning more than relevant [13,14].

IBD can have various symptoms, complications and consequences that may affect one’s sexual life [8,12], body image and intimacy [15] and sexual function [7,9–11], and a significant number of people with IBD do perceive these negative impacts on their sexuality. Chronic conditions, such as depression, fatigue, and sleep deprivation, can affect the sexual satisfaction of patients with IBD, even during periods of remission [16].

In patients with IBD, there is an increased risk of sexual dysfunction, particularly in females and in those with active disease or perianal disease [7,11]. Additionally, people with IBD exhibited lower performance in certain aspects of sexual function compared to those without the condition. In males, these included erectile function, satisfaction and quality, orgasm and desire performance, while in females, this covered pain, desire, arousal, lubrication, orgasm and satisfaction and quality [17]. Previous studies have identified that sexual/erectile dysfunction is not necessarily related to objective disease state and, more likely, is related to IBD symptoms [18] and psychological aspects [19].

A study by Riviere, Zallot [19] found that sexual dysfunction is highly prevalent among people with IBD: more than 40% of the men in the study had erectile dysfunction, and over half of the women experienced sexual dysfunction. The sexual dysfunction in men was linked to depression, while in women, anxiety, fatigue and poor quality of life were associated with impaired sexual function. Despite similar sexual activity rates to controls, people with IBD did not experience the same level of satisfaction in their sexual relationships compared to the general population.

Female gender and IBD surgery are risk factors associated with a negative view of body image, libido and sexual activity frequency. Moreover, those who have undergone limited resection are more likely to report adverse effects of IBD on these aspects of their life [20]. Sexual dysfunction following pelvic surgery may stem from anatomical nerve injury. Yet, it can also manifest as a consequence of the psychological effects of altered body image, sexual self-esteem, and relationships and intimacy [21]. Active disease is associated with decreased fertility, and paternal or maternal IBD increases the risk of IBD development for the offspring [11]. Studies have shown that women with IBD have fewer children or decide not to have children, a phenomenon referred to as ‘voluntary childlessness’ [22–24]. Voluntary childlessness is a complex concept that affects a woman’s life [25] and can be associated with stigma [26]. Patients with IBD, particularly with CD, are more likely to choose voluntary childlessness than healthy controls [11].

Marin, Manosa [27] found that half of women and one-third of men with IBD who were sexually active at the time of IBD diagnosis perceived that the disease negatively influenced their sexual life and that females with IBD have a higher risk of impaired sexual function than the general population. Women with IBD had a significant negative impact on body image, sexual activity and libido. Women were more likely to experience sexual inactivity, perceive an adverse effect of IBD on their sexual life and have body image concerns.

In the context of IBD, psychological factors (mood disorders, especially depression) are commonly reported as a prevalent psychological comorbidity and a significant contributor to sexual dysfunction [17,28–30].

### Objective

The purpose of this study is to investigate the perceived impact of Inflammatory Bowel Disease (IBD) on sexual life and intimate relationships, to contribute to the existing literature and to enhance understanding of the complex interplay between IBD, sexuality, intimacy and family planning, to foster a better quality of life for those living with this condition.

## Materials and methods

The data presented in this study were drawn from a broader study focusing on the experiences of people living with IBD. This study focuses on sexuality and family planning using qualitative analyses. This study followed the Standard for Reporting Qualitative Research (SRQR) guidelines to present the methodology and findings. This approach ensures qualitative research transparency, rigour, and clarity [31].

### Design

From May 2017 to April 2023, 36 in-depth, semi-structured interviews were conducted with 19 individuals with Crohn’s disease and 17 with ulcerative colitis living in the Czech Republic. The study used the DIPEx method (Database of Personal Experiences of Health and Illness) developed by the Health Experiences Research Group at the University of Oxford. It involves rigorous analysis of narrative interviews of people with particular conditions chosen to represent the broadest range of experiences [32]. The methodology has been standardized and adapted to the conditions of the Czech Republic [33]. This research design is used to study the experiences of people with various illnesses and disabilities [34,35]. The interviews were about the experiences of people living with IBD in general (diagnostics, medical and professional care, coping strategies, everyday life, etc.). The interviews were systematically coded.

### Study setting and recruitment

Participants were recruited through Czech organizations working with the target group (Pacienti IBD, ILCO – Voluntary Association of Ostomies), an Advisory Panel and support groups, including virtual communities on social networks. Most participants were recruited personally through direct referrals from gastroenterologists and other patients. This personal approach ensured a high level of trust and willingness to participate. To reach a diverse group of participants, advertisements were placed in online forums, social media platforms, and newsletters targeted at individuals with IBD. Recruitment materials emphasized the study’s focus on exploring personal experiences with IBD.

Participants were screened to ensure a diverse and representative sample, focusing on achieving maximum variation in the sample. This included consideration of factors such as age, gender, geographical location, stage of disease, type of IBD (Crohn’s Disease or Ulcerative Colitis), relationship status, and parental status. Participants were classified as ‘recently diagnosed’ if they had been diagnosed within the last two years. This classification was essential to capture a wide range of experiences from different stages of the disease.

Maximum variation was defined before the data collection by the DIPEx methodology [36,37]. The maximum variation sampling aimed to include a broad spectrum of experiences by selecting participants from various demographic backgrounds and disease conditions. This approach ensured that the study captured diverse perspectives, providing a comprehensive understanding of the impact of IBD on intimacy and family planning.

Interviews were conducted in person at the participants’ homes (24), at the researcher’s office (5), and places chosen by the participants according to their preferences (6). One interview was conducted online. Repeat interviews were not conducted.

### Inclusion and exclusion criteria

The inclusion criteria for the study required participants to be adults aged 18 and older with a confirmed diagnosis of Inflammatory Bowel Disease (IBD), specifically CD or UC, residing in the Czech Republic. They had to provide written informed consent and be able to speak and understand Czech. Exclusion criteria involved individuals with major comorbidities that could influence their IBD experiences, those unable to communicate effectively due to cognitive impairments or severe physical illness, and any potential participants who did not provide informed consent. Furthermore, participants were purposively selected to fulfil the requirements of a maximum variation sample.

### Data collection

The data were collected using the in-depth interview method, consisting of a narrative and a semi-structured part (relevant questions shown in Table 1).

**Table 1.** Part of the interview guide related to sexuality and family planning.

Part of the interview	Questions
1. Narrative part	Please tell me your story with IBD from the first symptoms to the present day.
	(After the participant finishes their narrative, the interviewer asks about topics they would like to clarify or expand on.)
2. Semi-structured part – questions where codes related to sexuality or family planning appeared	What is daily life like with such a disease?
	How does it affect your family life?
	How does your illness affect your relationship with your partner?
	How does it affect your intimate life?
	What has the disease taken from you, and what has it given you?

### Data analysis

The interviews were digitally recorded, then transcribed verbatim (by students trained in transcribing interviews and signed confidentiality agreements) and checked by the researcher (HB) for the correctness of the transcription; the anonymized transcripts were then sent to the participant for approval.

The data were then coded systematically in the NVivo software. Initially, the primary researcher (HB) independently coded five transcripts to develop an initial coding framework. This framework was collaboratively reviewed and refined through discussions with the entire research team, allowing for diverse insights and ensuring the framework’s robustness. After establishing the framework, HB, ZM, and KG coded all the transcripts independently. To ensure reliability and consistency, cross-coding was applied across these transcripts. Any new topics or themes that emerged during the coding process were discussed within the team and integrated into the existing coding framework, ensuring a dynamic and comprehensive analysis. All coding decisions and changes were meticulously documented in a shared coding diary, providing transparency and facilitating reflexivity among researchers.

The thematic analysis identified patterns and themes relevant to the study objectives. This approach involved constant comparison across different cases to identify similarities and differences. Deviant cases were also explored to provide a nuanced understanding of the data [38,39]. The interviews were read repeatedly, and individual statements were classified into thematic codes and then categorized. The categories were combined into particular topics through thematic analysis.

The ‘one sheet of paper’ (OSOP) technique [32], in which the researchers analyzed all sections of data that fall under codes ‘sexuality’, ‘body image’, ‘discomfort’, ‘partnership,’, ‘relationship’, ‘intimacy’, family planning’, ‘family’, ‘doctor’ and ‘messages’ was used. For this, researchers compiled relevant data on individual sheets with the participant’s ID, facilitating the identification of common elements and differences across participants. This technique helped synthesize complex information into coherent themes.

Analyses were carried out under supervision (by PT) by three researchers (HB, ZM and KG) trained in the DIPEx method. The interviews and the analyses were conducted in the Czech language. The results were then translated into English for this study (The DeepL translator was used for translations).

### Ethical considerations

The study was conducted according to the ethical principles outlined in the Declaration of Helsinki [40], and the Ethics Committee of the Olomouc University Social Health Institute approved the study on 22 May 2017 (approval n^o^ 2017/06). The risk of identification of participants from the included identifiers was weighed against the interpretative value they add, evaluated by the research team and ethics committee and was deemed negligible based on the high prevalence of the disease in the target country. The participants could clarify some details or delete part of the interview. All participants provided written informed consent to use the data for research and for publication. Participants were informed about their right to withdraw participation in the study at any time. Oral and written informed consent was obtained before the interviews were conducted. All information related to the interviews is confidential and protected during the publication process. Identifiable data were stored on a secure research server at the university. Only KG and HB have access to the non-anonymized data.

### Rigour and reflexivity

Following data interpretation using reflexive thematic analysis, the investigators’ subjectivity is a source to assist in knowledge production within the study [38]. As researchers bring their pre-understandings, expectations, knowledge and experiences to the research process, reflexivity and replicability are essential to qualitative research. Most interviews were conducted by HB, with seven interviews conducted by a student under guidance and supervision. HB is a psychologist with a focus on the psychological aspects of patients with IBD. Data analysis was conducted by the first author (KG). This social worker has previously been involved in several social health research projects, including research using the DIPEx methodology and ZM (psychotherapist and researcher). All researchers were trained in qualitative research and had no previous relationship with the participants. Furthermore, the project enlisted the support of medical supervisors who served as professional guarantors. These supervisors were three gastroenterologists, along with an IBD nurse. Additionally, other esteemed Czech experts, such as the director of the patients’ organization, actively contributed to the preparation and conducting of the research. Their involvement spanned all project phases, ensuring professional integrity and objectivity. Regular interactions occurred throughout the project, with three in-person meetings called the Advisory Panel. The Advisory Panel also included researchers from the Olomouc University Social Health Institute and two people living with IBD, one male and one female, representing the lay perspective. To maintain scientific rigour, an academic supervisor (PT), an experienced researcher in psychology and health, meticulously examined all the data and engaged in ongoing consultations and discussions throughout the research and analysis process. As for qualitative validation, we used member-checking triangulation of the data from transcripts and observational notes and compared these with disconfirming evidence [41].

## Results

### Characteristics of participants

The final sample consisted of 36 individuals with IBD. The sample was composed so that the participants came from different regions of the Czech Republic and municipalities of different sizes. They were selected to be at different disease stages and have experience with various medications and treatments. The demography of the participants is presented in Table 2.

**Table 2.** Sociodemographic factors.

Participants	*N*	%
men	16	44.4
women	20	55.6
Age		
20–29	11	30.6
30–39	9	25.0
40–49	7	19.4
50–59	5	13.9
60–69	4	11.1
Diagnosis		
Crohn’s disease	19	52.8
Ulcerative colitis	17	47.2
Age at diagnosis		
10–19	13	36.1
20–29	10	27.8
30–39	5	13.9
40–49	3	8.3
50–59	4	11.1
60–69	1	2.8
Education		
Education graduate	6	16.7
Secondary school diploma	13	36.1
University	17	47.2
Economic activity		
Employed (full-time)	13	36.1
Employed (part-time)	7	19.4
Economically inactive	10	27.8
n/a	6	16.7

*Note*: *N* = 36; mean 39.9; median 38; SD = 13.6.

### Findings

The main topics related to sexuality and family planning identified by thematic analysis were sexual activity, body image and discomfort, relationships, family planning and the role of the gastroenterologist in family planning decisions. As shown in Table 3, only some of the topics occurred in every interview, given the semi-structured interview scenario. In this part, we present findings from analyses of those topics.

**Table 3.** Occurrence of topics.

Participant	Sex	Age	Family status	Number of children	CD/UC	Surgery(ies)	Topics				
							Sexual activity	Body image and discomfort	Relationship	Family planning	Gastroenterologist
IBD01	M	31	Married	0	UC	No	x		x		
IBD02	F	58	Married	2	CD	Yes	x	x			
IBD03	M	40	In partnership	0	UC	No			x		
IBD04	F	66	Married	2	CD	No					
IBD05	F	38	Single	0	UC	No	x		x	x	x
IBD06	F	26	Single	0	UC	No	x		x		
IBD07	F	24	Married	1	CD	Yes		x	x	x	x
IBD08	M	29	Single	0	CD	Yes	x		x	x	
IBD09	F	49	Married	2	CD	Yes	x	x	x	x	x
IBD10	M	35	Single	0	CD	Yes	x				
IBD11	F	23	Single	0	CD	Yes		x	x		
IBD12	F	55	Married	2	CD	Yes	x		x		
IBD13	M	31	Single	0	CD	Yes	x	x			
IBD14	F	53	Married	1	CD	Yes			x	x	x
IBD15	F	28	Single	0	UC	Yes	x		x	x	
IBD16	F	35	Married	2	CD	Yes	x	x			
IBD17	F	26	In partnership	1	CD	Yes	x		x	x	x
IBD18	F	39	Married	1	CD	Yes		x	x	x	x
IBD19	F	52	Divorced	0	UC	Yes		x			
IBD20	F	28	In partnership	1	UC	No			x		
IBD21	M	42	Married	2	UC	Yes	x	x	x		
IBD22	F	21	Single	0	CD	Yes					
IBD23	F	43	Married	1	UC	No	x				
IBD24	M	30	Single	0	UC	No		x	x		
IBD25	M	41	Married	0	CD	Yes			x		
IBD26	F	25	Single	0	CD	Yes					
IBD27	F	28	Divorced	1	CD	No					
IBD28	M	28	Married	0	CD	Yes					
IBD29	M	36	Married	0	UC	No					
IBD30	F	67	Married	3	UC	Yes	x		x		
IBD31	M	67	Married	3	UC	No	x	x	x		
IBD32	M	40	Divorced	0	CD	Yes		x			
IBD33	M	69	Divorced	2	UC	No		x			
IBD34	M	43	Married	2	UC	Yes	x		x		
IBD35	M	38	Divorced	0	UC	No	x		x		
IBD36	M	52	Single	0	UC	No					

*Note:* M: male; F: female; CD: Crohn’s disease; UC: ulcerative colitis.

### Sexual activity – occasionally, sexual life had to be put on hold

In the context of Inflammatory Bowel Disease (IBD), perceptions of sexuality varied among participants. While some individuals reported never experiencing any issues in this area, others mentioned that their sexual experiences differed across different stages of the illness.

I don’t think it (IBD) would limit me in any way in my intimate life, maybe sometimes, but rarely. (IBD10)

During the acute phase of the disease, some participants experienced a reduction in their sexual activity.

What about sex? That’s zero so far. It’s not possible yet. If you move, immediately flatulence, winds. Crazy, terrible, I don’t give a damn. (IBD02)

For specific individuals, the impact of IBD on their sex life was occasional, and they mentioned feeling unwell to the extent that it negatively affected their intimacy. One female participant even reported that sexual intercourse was not feasible for her due to a recently diagnosed illness. However, there was hope for improvement over time, as medical professionals expected a gradual amelioration in her condition.

I don’t think it was so bad that it affected my intimate life or anything like that. Basically, for me, it’s kind of paradoxical that I have the greatest frequency of those stools, maybe more in the morning and afternoon hours, and then in the evening and afternoon hours, my bowels calm down, and there’s just, like, nothing happens at all, and it’s fine. So that’s why I’m building on that partner life, on those evenings, and that way, the problem just wasn’t there; of course, when I was a little bit worse when I knew that I was going more often that day, I’m like that, so my partner didn’t have any problem with that. It’s always a negotiation. (IBD09)

The participants’ sexual activity was notably affected by their overall health status. Physical discomfort, pain, scars, stomas or other medical interventions associated with IBD hindered some people from engaging in sexual activities. Additionally, technical problems during intercourse were observed in cases involving stoma.

Does the disease affect my intimate life? I think it’s different for everyone. But with the stoma, I understand the problems. I struggled with that at the time. When I had it, I wondered how it would be. I didn’t try it. (IBD13)

### Body image and discomfort – breaching barriers I would rather not cross

Moreover, the experience of intimacy was affected for some participants due to discomfort, such as feelings of flatulence and frequent bowel movements, which disrupted the sense of closeness with their partners.

Today, I laugh about it, but back then, it wasn’t funny at all. It’s not easy to explain to a new boyfriend that you go to the bathroom five times during a date. (IBD24)

In men, the worsening of body image only appeared in connection with an artificial intestinal stoma; otherwise, they mostly complained about feelings of shame related to disease manifestations, such as frequent stools or flatulence.

Of course, it takes away your physical strength, first of all. Secondly, bowel problems are not just stool problems. It’s also flatulence, which, just unfortunately, you can’t always do it where you’re supposed to do it. And this affects intimate relationships, yeah. (IBD31)

Furthermore, some women with IBD faced additional challenges, such as the use of corticosteroids leading to swelling and weight gain, contributing to a diminished sense of well-being and possibly impacting their sexual confidence. In addition to the challenges faced by some women in their intimate relationships and sexual life, the impact of IBD on body image was a notable concern. The physical symptoms, such as weight fluctuations, visible scars from surgeries and changes in body shape, contributed to feelings of self-consciousness and dissatisfaction with their appearance. Therefore, some women reported struggling with body image issues, feeling ashamed, less attractive or desirable due to the visible (and audible) effects of the disease and its treatments. They reported that these negative perceptions of their bodies led to decreased self-esteem and confidence, influencing how they perceived themselves in romantic and sexual contexts.

Now I’m going to take corticosteroids, my face is going to swell up, I’m going to gain 10 kilos, and my guy’s going to be like: Jesus Christ, what do I have next to me? (IBD07)

Coping with these body image concerns added an extra layer of complexity to the participants’ overall well-being and their ability to engage in fulfilling intimate relationships.

### Relationship – a partner needs to comprehend

In exploring the dynamics of partner relationships, it became evident that IBD could have contrasting effects. Some couples reported feeling divided by the illness, while others found that it strengthened their bond.

On one hand, it strengthened us (me and my boyfriend), but on the other hand, I feel like it kind of broke us a little. It’s also not easy for him. You are at an age where they should be thinking about starting a family, living together, managing finances, and so on, but instead, he has to visit his girlfriend in the hospital. But on the other hand, it’s also hard for me to be a support for him, because I’m often just trying to take care of myself, so this is also quite difficult. (IBD15)

For some participants, the experience of coping with the disease allowed them to communicate more openly with their partners about intimate matters, breaking down barriers and fostering a more profound sense of connection and support.

I’ve talked to women who have an ostomy, and they haven’t had a problem. But they were like, ‘Well, you guys have it easier’. But I think maybe the women have it easier. If the woman’s a bit more outgoing, chick, then maybe her man doesn’t care so much that she has an ostomy. But it really seems to me that some of these women, I just think it would be a very big problem for them to have a guy with an ostomy. (IBD21)

### Family planning – seeking the suitable moment for a baby

Family planning emerged as a crucial topic for some of the women who participated in the study, especially for those whose disease manifested before having children. Men in our study did not express such concerns. In some cases, physicians advised against starting a family due to the patient’s health status. This recommendation occurred both in cases where the participant had been diagnosed long ago and in instances where the participant was recently diagnosed and is currently experiencing an acute phase of the disease. One woman attributed the medical reservations towards her having a child to the lack of sufficient information about IBD and pregnancy in a socialist society two decades ago.

Then I got myself together enough that I convinced my husband that we could have a child, which they initially didn’t recommend. Well, at first it didn’t work naturally, so we tried IVF, which also didn’t succeed. But I told myself I wouldn’t give up, and within a year, I got pregnant naturally. (IBD14)

Approaches to family planning varied among participants. Some did not consider their condition during pregnancy planning and did not plan their pregnancy. In contrast, others sought information and consulted with their physicians to adjust medication and identify the most appropriate time to start a family.

Some women hesitated to conceive and waited until their disease reached a remission phase before proceeding with pregnancy. No women in our study opted for voluntary childlessness.

IBD affects a person a lot in family planning. Some people just say: Let’s get married, and that’s it. Then we’ll have kids one day, and boom. But when you have colitis, you have to more or less plan, ‘Well, I’m fine now, so we can get married in about two months before I have another flare-up’, for example. The same is true with the baby, which can often be problematic. For me, for example, after these surgeries, they say that there is a three-fold increased risk of infertility, so even that is kind of quite unpleasant, but it affects you afterwards. (IBD15)

Several women discussed their experiences with contraception while managing IBD. Hormonal contraception, in some cases, was not recommended due to absorption concerns, leading them to opt for non-hormonal methods. One participant mentioned that her partner experienced discomfort (did not like the barrier method of contraception) as a result of this decision. Another participant reported being unable to take hormonal contraception due to her health condition – specifically, worsened liver tests – but remained uncertain about any direct correlation with her IBD.

Some women expressed concerns about the potential risks of medication or their underlying disease on the foetus, fearing that physiological changes during pregnancy could trigger worsening disease symptoms. For those who had undergone medical procedures, there were also apprehensions about complications arising due to the expanding pregnant abdomen.

According to what’s written on all those medications, it shouldn’t be a problem, and according to what various girls have posted on social media, no one has had any problems with it. So, getting pregnant is OK. And that would be more like a problem with the disease itself; you either get worse or better. That’s hocus pocus. Nobody tells you that in advance, either. (IBD05)

### Role of the gastroenterologist – collaborating on pregnancy plans with my gastroenterologist more than with my spouse

Women appreciated the support of physicians when they decided to have a baby, but others felt that their doctors had concerns about their health status. Some women actively engaged in extensive planning for their future pregnancies with the assistance of their gastroenterologist.

And when I eventually brought my daughter to my doctors, they nicknamed her after my doctor’s name, saying that she was like our joint creation. He got me to where I am now, where I was able to have my child. (IBD17)

Women sought to find the optimal time for conception, considering the course of their disease.

It’s definitely really important to discuss this with the gastroenterologist. I needed to clearly know when I would reach a stage where I wouldn’t endanger the fetus in any way. All I wanted to know was: Just tell me how long it will take before I can conceive. And my partner was put in a position: It’s now or never. (IBD07)

They adjusted their medication regimen and underwent various examinations, such as blood tests, to ensure the best possible conditions for pregnancy.

I’ve had many discussions with gastroenterologists, multiple gastroenterologists. I wanted to be sure to have a second opinion. They took my blood, so they knew that the half-life of the corticosteroids takes a while, so they could tell: Yes, now you’re clear, now you’re not going to endanger the foetus, you can start. (IBD07)

The participant’s experience underscores the need for gastroenterologists to play a more active and empathetic role in the family planning process. Without clear guidance from their primary IBD specialist, patients may struggle to make informed decisions about pregnancy, potentially leading to unnecessary stress and anxiety.

And when I asked my doctor at the gastro clinic about it, he looked at me and said: ‘You don’t seem to read my reports, you don’t remember what I told you last time? You want to be pregnant?’ He was basically making fun of me. Then, instead of telling me the pros and cons, whether I can or can’t, he said, ‘Well, maybe it would be better if you waited until you stop taking these particular medications’. (IBD05)

Some women who planned their families several decades ago encountered more concern from doctors about a potential pregnancy.

When I came to the doctor and said that I was pregnant, without saying anything else, this doctor immediately suggested that it wouldn’t be a problem to issue a certificate for termination due to my diagnosis. I don’t know if at that time there was really little experience with it, or if women were explicitly being discouraged from pregnancy back then, it’s hard to say; it was a transitional period, and the world was still closed off from us. (IBD09)

## Discussion

This study explored the impact of IBD on the sexuality and family planning of people living with IBD. Qualitative analyses revealed five main topics: sexual activity, body image and discomfort, partner relationship, family planning and the role of the gastroenterologist in family planning decisions. Participants’ perceptions of sexuality varied, with some reporting reduced sexual activity during acute phases of the disease. Physical discomfort, medical interventions and technical issues (when having a stoma) affected sexual activity for some individuals. Intimacy and body image were also disrupted by discomfort and self-consciousness related to IBD symptoms. IBD had contrasting effects on partner relationships, with some couples feeling divided by the illness while others reported increased communication and support. In women, family planning was a notable concern. They used various approaches to family planning, seeking information and support from their gastroenterologists. Women expressed concerns about the potential risks of medication and physiological changes during pregnancy and appreciated the support and collaboration of their physicians in making pregnancy decisions.

The study included young participants but also participants aged over 50, some of whom may have reflected on family planning and reproductive decisions made 10–20 years ago. Their experience differs from the experiences of younger participants, who face a contemporary healthcare environment and societal norms. This contrast highlights the evolution of IBD management and family planning support and underscores the importance of considering generational differences when interpreting the findings. Over the past two decades, significant changes have occurred in the management of Inflammatory Bowel Disease (IBD), including advancements in medications and treatment approaches [42–44]. These developments probably improved the quality of care and outcomes for patients, influencing their experiences with family planning and pregnancy. Additionally, societal norms regarding family planning have evolved, reflecting broader shifts in attitudes toward reproductive health and patient autonomy [45–47]. This changing landscape is reflected in the narratives of our participants, particularly those who have lived with IBD for many years. For example, some participants highlighted a lack of information and support regarding IBD and pregnancy in earlier decades, contrasting with the more comprehensive resources available today.

### Sexual activity

Some, but not all, participants in our study experienced difficulties in the area of sexuality due to their illness. The majority of previous studies warn about the negative impact of IBD on sexual life [14,15]. In the study of Barros, Alencar [8], 50.5% of patients stated that the disease did not affect their sexual life, while 9% reported that they chose sexual abstinence due to the impact of the disease. The IBD symptoms that interfered most significantly with their sex life were abdominal pain (40%), flatus (28%) and weakness (24%). Despite this, 67.7% of patients reported reasonable sexual satisfaction [8]. In a study by Marin, Manosa [27], the primary complaint associated with a deterioration in sexual life was fatigue. Participants in our study discussed a reduction in sexual activity primarily due to fatigue and worsened health conditions. However, many did not report any impact of the disease on their sexual activity. Unlike other studies, our results are not as alarming. This contributes to the theory that rather than physiological limitations, the reason for the deterioration of sexual health in many patients are psychological factors, which have been mentioned in other studies as well [20].

### Body image and discomfort

Our findings regarding the adverse effects of a damaged body image and discomfort on sexual life and intimacy align with previous research in this field [15,48]. People living with IBD experience socially embarrassing symptoms, including diarrhoea, rectal bleeding, urgency, incontinence, bloating and distension, borborygmi and flatulence, and may also experience disabling skin and joint disease, malnutrition, pain and fatigue. The primary factors influencing dissatisfaction in individuals with IBD are disfigurement and loss of function resulting from surgery, steroid use and disease activity, rather than the specific type of disease or its severity [48]. Collateral effects of corticosteroids, surgical scars and body thinness were noted as critical issues affecting their body image [27]. Individuals with an ostomy, in particular, experience a distinct decline in body image [49]. This is a topic that should be addressed by specialists (i.e. gastroenterologist, IBD nurses), mainly because individuals with IBD who have a poor body image experienced lower sexual satisfaction and overall quality of life [48].

### Relationship

Muller, Prosser [20] reported that over half of the patients felt that IBD had affected their relationship status. In contrast, our study indicates that the influence of IBD on relationships can often be positive. This was evident through comments expressing how the disease brought them closer to their partners. Many women expressed gratitude for their understanding partners, who provided support. The significance of having an understanding social environment, especially a supportive partner, in coping with IBD has been consistently observed in various studies, particularly concerning adjusting to an ostomy [49]. Informing one’s partner of the diagnosis helps to understand disease limitations, including symptoms like abdominal pain, dyspareunia and perianal issues (fistulae, fissures or abscesses). Partner support is crucial for fostering positive attitudes towards sexuality and body image [49].

### Family planning

In our study, certain women expressed concerns about waiting for the ‘right moment’ to get pregnant and were worried about the health of the foetus. This aligns with the advice from Szymanska, Kisielewski [50], which recommends planning pregnancy during the remission phase of IBD. Moreover, it is reassuring to note that most of the drugs used in IBD treatment are considered safe for both the mother and the foetus, as mentioned in the same study [50].

For women with IBD, it is worthwhile to plan the right timing of conception when deciding to have children. The pre-pregnancy counselling to address parental concerns, to aim for disease remission, and to discuss medication use during pregnancy is also recommended by the European Crohn’s and Colitis Guidelines on Sexuality, Fertility, Pregnancy, and Lactation [50]. Therefore, choosing the appropriate contraception is an important topic for many couples. The ideal contraceptive should have a very low to no failure rate and should not significantly impact IBD disease activity. The efficacy of oral contraceptives does not seem to be reduced in women with IBD [50] and they are generally safe for women with IBD [51], with progestogen-only options being preferred to lower the risk of venous thromboembolism [52]. However, the effectiveness of oral contraceptives can be reduced in cases of extensive ileal Crohn’s disease and multiple abdominal surgeries due to ileal malabsorption [53].

Interestingly, nobody mentioned the phenomenon of ‘voluntary childlessness’ in our study. Inactive IBD has little to no impact on fertility rates, but psychological comorbidities and misconceptions about the disease are assumed to lead to voluntary childlessness among people living with IBD [24]. A study by Purewal, Chapman [54] suggests that 19% of IBD women choose to remain voluntarily childless [54], and this decision is more common among those with inadequate knowledge about family planning and pregnancy. Pre-conception counselling, ideally initiated at the time of IBD diagnosis, significantly reduces the likelihood of voluntary childlessness [23,54]. A study focused on the association between family planning and awareness in the Czech environment would be highly valuable for a more profound comprehension of this phenomenon. It is possible that participants in our study received sufficient information to ensure that the illness did not influence their decision to have a child if they desired one. However, it was observed that they postponed the decision until their health condition stabilized.

### Gastroenterologist’s role in family planning

It is encouraging to observe that for women in our study, one of the main topics was the role of gastroenterologists in family planning, and they perceived professional counselling as substantial support. Discussion with a physician, especially a gastroenterologist, may influence IBD-specific reproductive knowledge and (as already explained) reduce the voluntary childlessness rate [23], and regular check-up visits before conception, during pregnancy and after delivery decrease the risk for woman and foetus and eliminate unnecessary fears [50]. During the consultation, the attending gastroenterologist and IBD nurse should actively inquire about these issues since IBD-related impairments extend beyond the gastrointestinal tract, and people with IBD may not readily share psychological or sexual concerns without being prompted [55]. This topic deserves more consideration because patients are not comfortable discussing their sex life and personal relationships with their physicians [56]. Replicating studies about perceived taboo in the Czech environment would be of great interest. Rosso, Aaron [10] recommend that people with IBD should have early discussions with their healthcare team about their pregnancy plans, including contraception, before conception, during pregnancy, and that after birth, IBD nurses must provide guidance and information to people with IBD and their partners. If at all practicable, discussions about pregnancy should begin before conception [10]. People with IBD will likely need to talk with other medical professionals. Research has demonstrated the vital role of IBD nurses in offering information and support to help people living with IBD navigate the decision-making process effectively [57,58]. This aspect holds considerable importance within the clinical practice of the Czech Republic. The role of gastroenterologists, but also of IBD nurses, is crucial, as they can engage with patients for more extended periods in less formal settings, particularly during the administration of biological treatment or patient education.

### Strengths and limitations of the work

Our study is the first of its kind in the Czech Republic, exploring the impact of Inflammatory Bowel Disease (IBD) on sexuality and family planning with IBD. This novelty contributes to filling a research gap in this area. The study utilized the DIPEx method, a well-established and rigorous approach to analyzing narrative interviews. This methodology ensured consistency and credibility in the data analysis process. By employing qualitative analyses, we gained in-depth insights into experiences, perceptions and challenges related to sexuality and family planning of people living with IBD. This approach allowed us to capture rich and detailed narratives from participants. We included a diverse group of participants with both Crohn’s disease and ulcerative colitis, which allowed us to capture a broad range of experiences and perspectives on the impact of IBD on sexuality and family planning. The study’s findings have practical implications for healthcare professionals, highlighting the need for improved communication, support and guidance for people with IBD in addressing intimate and family planning concerns. It also contributes to the importance of the need for proactive information dissemination and open communication from professionals. However, several limitations of this study must be considered when interpreting the results. The sample of participants was heterogeneous, reducing the qualitative analysis’s scientific rigour. Some participants grounded messages on long-term medical care experience, so they may not be relevant for those diagnosed more recently when some medical procedures have already been innovated. Participants were from different regions and circumstances, with different disease severity, ages, diagnosis age, and other sociodemographic factors. One limitation of this study is the age distribution of participants, which included a significant number of individuals over 50. This age group may have provided insights based on experiences from earlier decades. These historical perspectives, while informative, may not fully reflect current practices and experiences of those navigating family planning and IBD management in today’s healthcare context. Future research should consider these generational differences to enhance understanding across age groups. Another limitation of the sample was that we failed to recruit more participants from minorities and socially disadvantaged environments.

The interpretation of the results is also limited by the need for more objective information about the disease state of participants (e.g. CDAI score, SCCAI score), comorbidities and medication use.

### Recommendations for further research

In further research, it would be beneficial to complement our findings with a quantitative, ideally representative study in the Czech context, demonstrating our findings’ extent and generalizability. Future studies should aim to include more representation from minority groups, such as diverse ethnic and gender groups, and consider participants from various socioeconomic backgrounds, including education levels and financial status. Additionally, it would be valuable to include research from the perspective of healthcare professionals, focusing on their knowledge and views on sexuality and family planning with IBD. Investigating the role of IBD nurses in providing specialized support and guidance to people with IBD in managing their intimate lives and family planning decisions would also be of great value.

### Implications for policy and practice

Our study provides valuable insights into the challenges people with IBD face in their intimate lives and reproductive choices. It could improve understanding among healthcare professionals, fostering more informed and open communication with their patients. The findings could guide the development of targeted interventions and support programmes tailored to the specific issues related to sexuality and family planning for individuals with IBD.

## Conclusion

The study addressed the impact of Inflammatory Bowel Disease (IBD) on the sexuality and family planning of people living with IBD in the Czech Republic, which was previously an unexplored area of research. The study illuminates the challenges surrounding the intimate lives and reproductive decisions of people with IBD, emphasizing the necessity for proactive information dissemination and open communication from professionals. Furthermore, the study’s results could be utilized to develop targeted interventions and support programmes to address the specific issues related to sexuality and family planning for individuals with IBD.

## Data Availability

The data underlying this article are available from the corresponding author upon reasonable request.
